# Cadmium up Taking and Allocation in Wood Species Associated to Cacao Agroforestry Systems and Its Potential Role for Phytoextraction

**DOI:** 10.3390/plants12162930

**Published:** 2023-08-13

**Authors:** Donald A. Galvis, Yeirme Y. Jaimes-Suárez, Jairo Rojas Molina, Rosalba Ruiz, Fabricio Eulalio Leite Carvalho

**Affiliations:** 1 Centro de Investigación La Suiza, Corporación Colombiana de Investigación Agropecuaria (Agrosavia), Rionegro 250047, Santander, Colombia; 2 Facultad de Ciencias Básicas, Universidad de Córdoba, Montería 230002, Córdoba, Colombia

**Keywords:** agroforestry, cadmium toxicity, heavy metals, *Theobroma cacao*, phytoremediation

## Abstract

Trees in cacao Agroforestry systems (AFS) may present a high potential for cadmium (Cd) phytoextraction, helping to reduce Cd in cacao (*Theobroma cacao* L.) plants grown in contaminated soils. To assess this potential, four forest fine-woody species commonly found in cacao high-productive sites in Colombia (*Tabebuia rosea*, *Terminalia superba*, *Albizia guachapele*, and *Cariniana pyriformis*) were exposed to contrasting CdCl_2_ contamination levels (0, 6, and 12 ppm) on a hydroponic medium. Growth dynamics, tolerance index (TI), and Cd concentration and allocation in leaves, stems, and roots were evaluated for up to 90 days after initial exposure. *T. superba*, *A. guachapele*, and *C. pyriformis* were classified as moderately tolerant (TI > 0.6), and *T. rosea* was considered a sensitive species (TI < 0.35) under 12 ppm Cd contamination. Despite showing a high stem Cd concentration, *C. pyriformis* also showed the lowest relative growth rate. Among the evaluated forest species, *A. guachapele* exhibited the highest Cd accumulation capacity per plant (2.02 mg plant^−1^) but also exhibited a higher Cd allocation to leaves (4%) and a strong decrease in leaf and stem dry mass after 90 days of exposure (~75% and 50% respectively, compared to control treatments). Taking together all the favorable features exhibited by *T. superba* as compared to other CAFS tree species and recognized phytoextractor tree species in the literature, such as Cd hyperaccumulation, high tolerance index, low Cd concentration in leaves, and high Cd allocation to the stem (harvestable as wood), this species is considered to have a high potential for cadmium phytoextraction in cocoa agroforestry systems.

## 1. Introduction

In Colombia, there are around 188,722 hectares of cacao cultivated by 65,341 producer families. This product has won important international recognition for its quality, while showing a positive economic balance, increasing exports by up to 13% in the last 5 years [1]. However, despite an increase in demand for cacao and the great possibilities to promote cultivation, it has recently been observed that quality requirements have also increased, especially concerning Cd content. This is because the consequences of exposure to Cd on human health have already been reported [2,3], alerting international control agencies to define the maximum levels allowed in food, including chocolate and other cacao-derived products [4].

Various studies reveal an intimate relationship between Cd and cacao plants. Argüello et al. [5] reported that the average concentration of Cd in cacao beans from South America was almost three times higher than in Central America and East Africa and ten times higher than that found in West Africa. This previous report also suggested that high concentrations of Cd in soils from South and Central America could be of geogenic origin, unrelated to human contamination. On the other hand, Pabón et al. [6] reported that in Colombia, 57% of the samples of processed cacao beans exceed limits established by the European Community, and Bravo et al. [7] reported that 42% of the soil samples processed in Colombia exceed the threshold defined for the natural concentration of Cd in soil. 

Therefore, there is an increasing concern regarding the future of cacao exports in South America, especially in Colombia. To eliminate Cd from contaminated areas, the producers have successfully applied unconventional techniques involving biological processes. Indeed, phytoextraction technology has been widely used for mitigating contaminated soils. This technology is based on the ability of some plants to remove heavy metals from soils and accumulate them in the harvestable parts [8,9]. This approach is less expensive and harmful to the environment than conventional remediation systems [10,11]. In addition, it may allow producers to obtain other products, such as wood.

Cacao in Colombia is mostly grown under agroforestry systems (AFS), highlighting its environmental importance [12]. Indeed, cacao plants growing in agroforestry systems also play a crucial role in local ecosystems since they contribute to the fixation of atmospheric CO_2_, soil conservation, and the increase of general fertility [13,14,15]. The floristic composition in AFS varies according to the structure, proportion, and diversity of the shade arrangement. These features may also affect environmental conditions such as hydric dynamics. The most commonly used forest species in the shade canopies of cacao plantations in Cameroon are *T. superba, Spathodea campanulate* (P. Beauv.)*,* and *Albizia adianthifolia* (Schumach.) W. Wight [16]. Mercedes-Ordoñez et al. [17] reported a rich floristic composition in a cacao-producing area of Colombia, dominated by forest species in order of predominance: *A. guachapele*, *Erythrina poeppigiana* (Walp.) O.F. Cook, *Gmelina arborea* (Roxb.), and *Cordia alliodora* (Ruiz and Pav.) Oken. Among the species that associate the cacao agroforestry model with tropical fine-timber species, *Tectona grandis* (L. f.), *Swietenia macrophylla* (King), *T. rosea, Hevea brasiliensis* (Willd. ex A. Juss.) Müll. Arg., and *C. pyriformis* are highlighted [13]. Moreover, *C. pyriformis* was reported to allow cacao to maintain constant photosynthetic activity during the dry and wet seasons [18] and improve water use efficiency and carbon storage in association with specific cacao genotypes [19].

The phytoextraction hypothesis is based on the premise that plants classified as heavy metal hyperaccumulators can promote soil decontamination. Accordingly, the roots of the hyperaccumulator plant absorb the contaminants from soil or water, transport them, and accumulate them in the aboveground biomass such as shoots and leaves [20]. Specifically for cadmium, plants with concentrations above 100 ppm in aboveground tissues are considered hyperaccumulators [21,22]. Thus, different species of plants, both herbaceous and arboreal, have been studied for their phytoextractor potential, which relates to their ability to concentrate cadmium in above-ground tissues and general tolerance to heavy metal exposure [11]. *Salix* spp., Castor, Corn (*Zea mays*), *Populus* spp., *Jatropha curcas*, *Helianthus annuus*, *Arabidopsis helleri*, *Minuartia verna*, *Viola bashanensis*, *Cerastium arvense*, *Claytonia perfoliate*, *Thlaspi caerulescens*, *Brassica juncea*, *Arabidopsis thaliana*, *Liriodendron tulipifera*, *Morus alba*, *Solanum nigrum*, *Averrhoa carambola*, *Pteris vittate*, *Astragalus sinicus*, *Brassica oleracea*, *Raphanus sativus*, *Eichhorina crassipes*, *Euphorbia cheiradenia*, *Ricinus communis*, *Sedum alfredii*, and *Trifolium alexandrinum* are examples of useful plants for remediating cadmium-contaminated soils reported in the literature [11].

The heavy metal phytoextraction technology using forest trees, however, is more effective than those employing herbaceous or shrub species since trees exhibit a higher yield of biomass production [23,24]. In addition, trees have more developed root systems, access a larger area for decontamination, have a longer life cycle, can accumulate greater amounts of heavy metal, are adapted to different growth environments, and can sequester metals in the secondary growth process, in addition to promoting other associated ecosystem benefits [25]. Indeed, previous reports have evidenced that some tree species of the Salicaceae family can accumulate very high levels of heavy metals [26,27]. Indeed, some studies have focused on the remarkable potential of *Salix* sp. and *Populus* sp. in phytoextraction [28,29,30,31,32,33,34,35,36,37], showing that these species could be used as an efficient and cost-effective method to remove Cd contamination from agricultural soils [22,36]. On the one hand, the benefits associated with accompanying trees in cacao agroforestry systems have been widely studied in terms of carbon capture, water use efficiency and other nutritional benefits [18,19,38]. However, on the other hand, the potential use of different timber trees commonly associated with cacao AFS for cadmium phytoextraction strategies is still scarcely studied.

The aim of the present study is to prospect for cadmium phytoextraction characteristics in forest species commonly associated with cocoa agroforestry systems (CAFS). In order to reach this objective, we tested the hypothesis that the different forest species that are commonly found accompanying CAFS [18,19,38,39,40,41] have a significant variation in the dynamics related to Cd uptake, accumulation, allocation, and subsequent effects on plant growth and development. It is important to highlight here that the forest species used in the present study coincide with those that are most commonly found in CAFS already established in the studied area [18,19,39,40,42]. Thus, identifying phytoextraction characteristics in any of these species would be an important advantage to cadmium phytoremediation strategies since there are already large areas of this CAFS currently established in this territory. Accordingly, assessing these potential features of CAFS could provide a low-cost and effective solution for the excess Cd issue in cacao cultivation in South America.

## 2. Materials and Methods

### 2.1. Plant Material and Experimental Conditions

Plants were grown under greenhouse conditions at the Agrosavia “la Suiza” research center located in the department of Santander, Colombia. Certified seeds of the forest species *T. rosea, A. guachapele, C. pyriformis* were bought from the forest seed producer “El semillero S.A.S”. *T. superba* seeds were obtained from 14-year-old trees growing on the Agrosavia “la Suiza” research center, which were originally donated by FHIA (Fundación Hondureña de Investigación Agrícola). The seeds were germinated and grown first for 60 days in plastic trays containing peat, and subsequently seedlings were transferred to 30 L plastic containers containing Hoagland’s solution [43], where they were kept for up to 180 days. The concentration of dissolved oxygen in the medium was maintained between 9 and 11 ppm using blower-type oxygenator equipment with a capacity of 3 L h^−1^. pH, electrical conductivity, and total dissolved solids (TDS) were controlled daily using the Hanna multiparameter meter (Ref: Hi98129). The pH was regulated (5.8–6.2) by applying phosphoric acid (H_3_PO_4_) or hydroxide (KOH). The total dissolved solids (TDS) were always kept between 1500 and 2000 ppm. The electrical conductivity remained between 1 and 2 dS m^−1^. The Hoagland nutrient solution was used as a control treatment, and two treatments with concentrations of 6 and 12 ppm of Cd were used. The Cd exposure was carried out 90 days after sowing (referred to thereafter as day 0 of exposure) using cadmium chloride (SIGMA-ALDRICH, St. Louis, Missouri, United States, CdCl_2_, 99.99% trace metal basis). The standard Hoagland and Arnon’s hydroponic medium presented an initial Cd content of 0.134 mg kg^−1^, which was thereafter referred to as a “0 ppm” treatment.

### 2.2. Growth Analysis and Total Cadmium Content

Morphological growth parameters (plant height, basal diameter, and number of leaves or leaflets) were recorded at 60, 90, 120, 150, and 180 days after sowing (DAS). Three destructive samplings were carried out at 120, 150, and 180 DAS (30, 60, and 90 days after applying the cadmium treatments) to evaluate the concentration of the metal accumulated in roots, stems, and leaves. Total cadmium content was estimated employing spectrometry of Inductively Coupled Plasma Atomic Emission [44]. Before the analysis, fresh mass was recorded and subjected to oven drying for 48 h at 60 °C to determine the dry biomass accumulated by the different tissues. Relative growth rate (RGR) was estimated following the equation proposed by [45], RGR = [ln(W2) − ln(W1)]/(t2 − t1), where W1 and W2 are the initial and final dry weights (g) for different time intervals of cadmium exposure time (0–30, 30–60, and 60–90 days after exposure). The methodology proposed by [46] was used to estimate the tolerance index (TI = Total Plant Dry Mass presented under the cadmium treatment/Total Plant Dry Mass presented under the control treatment). Therefore, TI proves the occurrence or not of a dry mass decrease in plants treated with different levels of cadmium in comparison with their respective controls.

### 2.3. Experimental Design and Statistical Analysis

The experiment was arranged under a completely randomized block design. The factorial design of the treatments consisted of 3 × 4 × 4, with 3 doses of cadmium chloride contamination (0, 6, and 12 ppm), 4 forest species (*C. pyriformis, T. rosea, A. guachapele, and T. superba*), and 3 times of destructive sampling (0, 30, 60, and 90 days after the contamination). For each destructive sampling, a total of 3 replicates were used, each consisting of a fully independent plant (*n* = 3). In the specific case of non-destructive growth determinations, i.e., stem diameter, plant height, and number of leaves, 9 replicates were used, each one also consisting of 1 independent plant (*n* = 9), and an additional sampling at 30 days before the cadmium chloride application of cadmium was also performed, therefore compiling information from −30, 0, 30, 60, and 90 days of exposure. For each parameter evaluated, an ANOVA (*p* ≤ 0.05) was performed, and when significant differences were detected, a Tukey’s test (*p* ≤ 0.05) was performed to evaluate significant differences between treatments.

## 3. Results

### 3.1. Terminalia superba Showed Hypertolerance to Moderate Cadmium Contamination

**Figure 1:**
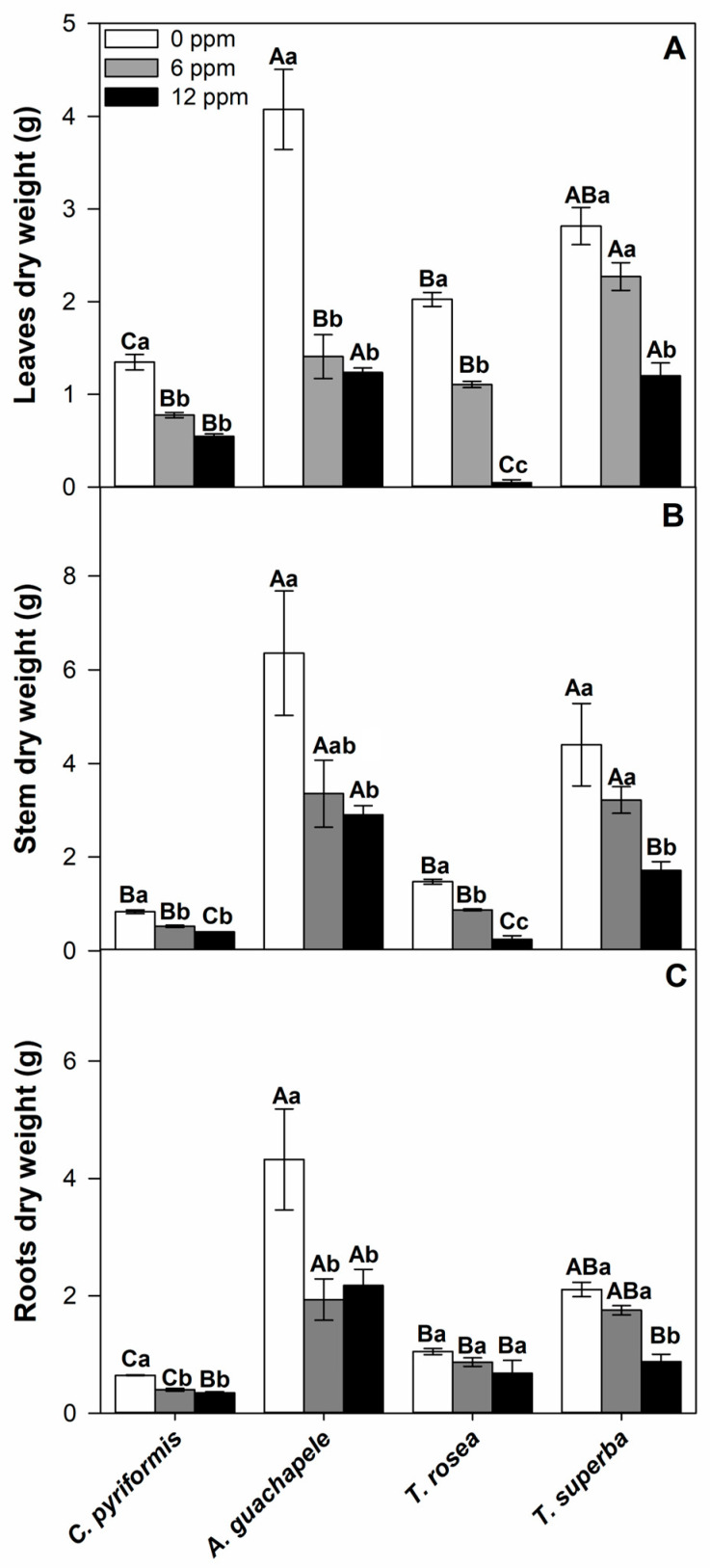
Dry mass in leaves (**A**), stems (**B**), and roots (**C**) of four forest species (*C. pyriformis, A. guachapele, T. rosea, and T. superba*) exposed to contrasting levels of cadmium (0, 6, and 12 ppm) for 90 days. Mean values ± standard error (*n* = 3). Different capital letters mean significant differences between plant species at the same contamination level, and different lowercase letters represent significant differences between cadmium treatments in the same plant species, according to Tukey’s test (*p* < 0.05).

Aiming to verify the phytoextraction potential in four different forest species commonly associated with ASF-cocoa, we first sought to evaluate crucial growth and development indicators in plants treated with 6 and 12 ppm of CdCl_2_. Under moderate levels of cadmium contamination (6 ppm), only *T. superba* was able to maintain the dry mass of roots, stems, and leaves without significant differences compared to the control treatment, which indicates that this species can be considered hypertolerant to moderate concentrations of cadmium (Figure 1, Table 1). Under extremely high concentrations of cadmium (12 ppm), however, the total dry mass of leaves, stems, and roots of all four forest species showed a downward trend. Regarding the dry mass of leaves, the species *T. rosea* suffered the most drastic reductions, reaching only 2.5% of the dry mass of leaves presented by the plants under control conditions after 90 days of exposure to 12 ppm Cd. Under the same cadmium contamination, *C. pyriformis*, *A. guachapele*, and *T. superba* presented, respectively, 41%, 30%, and 43% of leaf dry mass compared to their respective controls after 90 days of exposure (Figure 1A). A similar downward trend was observed in the stem dry mass of *T. rosea* under 12 ppm cadmium, which exhibited only 16% of the control stem dry mass, while *C. pyriformis*, *A. guachapele*, and *T. superba* had 48%, 46%, and 39%, respectively, of the stem dry mass of their respective controls (Figure 1B). Root growth, on the other hand, was less impacted by cadmium treatments, since under 12 ppm of cadmium, the dry mass of roots in *C. pyriformis*, *A. guachapele*, *T. rosea*, and *T. superba* showed respectively 54%, 50%, 65%, and 42% of their cadmium-free references (Figure 1C).

**Figure 2:**
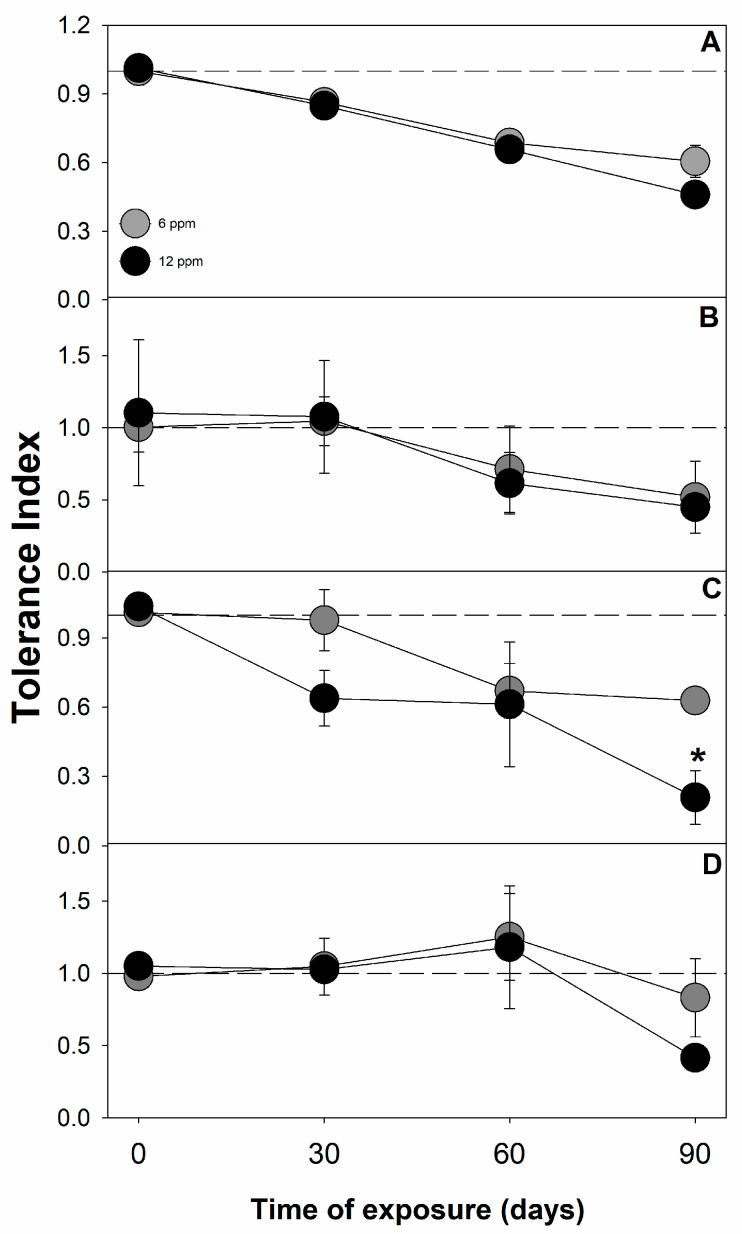
Tolerance index in *C. pyriformis* (**A**)*, A. guachapele* (**B**)*, T. rosea* (**C**)*, and T. superba* (**D**) exposed to contrasting levels of cadmium (6 and 12 ppm) for up to 90 days. Mean values ± standard error (*n* = 3). Asterisks represent significant differences between cadmium treatments in the same plant species and at the same time of exposure, according to Tukey’s test (*p* < 0.05).

Based on the changes in the total dry mass, an important indicator related to the physiological stress caused by excess cadmium in plants can be estimated, defined as the tolerance index (TI), which refers to the fraction of the total dry mass that is reduced in response to exposure to the contaminant. In line with the dry mass results, *T. superba* stood out again for presenting high TI values under conditions of 6 ppm Cd (approximately 0.8), which corroborates its hypertolerance potential under these conditions (Figure 2). On the other hand, *T. rosea* showed a significant difference in TI between 6 and 12 ppm, which was more than 50% lower at the higher cadmium concentration (Figure 2C). Regarding the relative growth rate (RGR), under a moderate level of Cd contamination (6 ppm), *T. superba* did not show significant differences in relation to the rates presented by the same species under control conditions (Table 1). Under 12 ppm, however, the relative growth rates were negatively affected, even in *T. superba*. Interestingly, under the contamination of 12 ppm, *T. superba* was able to maintain positive rates (real growth) up to at least 60 days of exposure. Under similar conditions, *T. rosea* showed negative rates from the first 30 days of exposure, which corroborates the greater sensitivity to stress caused by high cadmium in this species (Table 1). Furthermore, for all the plant species evaluated, non-destructive growth indicators such as stem diameter, plant height, and the number of leaves did not show significant impacts until 60 days of exposure to cadmium, regardless of the level of contamination (Figures S1–S3).

### 3.2. Terminalia superba Showed High Allocation of Cadmium to Stem Tissues

**Figure 3:**
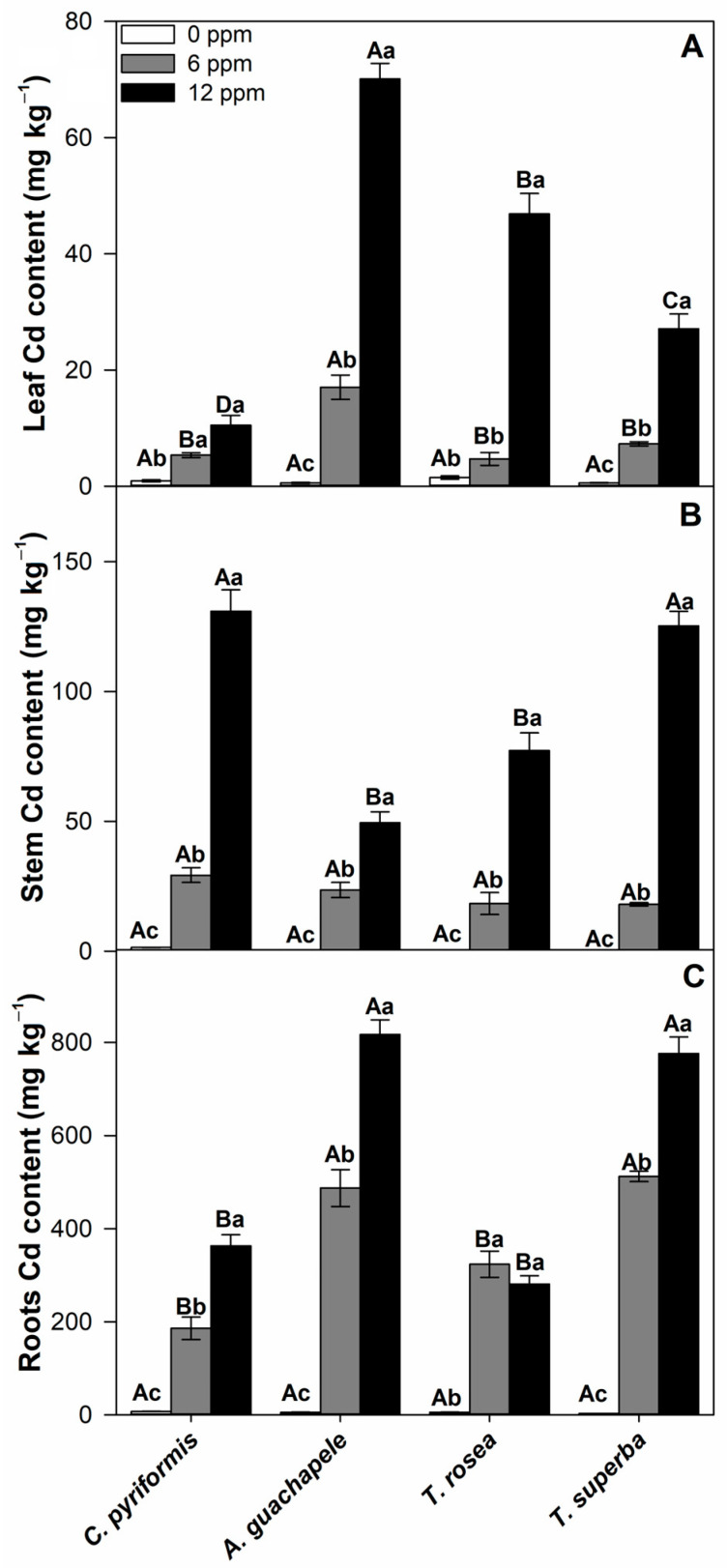
Cadmium content (mg kg^−1^ dry weight) in leaves (**A**), stems (**B**), and roots (**C**) of four forest species (*C. pyriformis*, *A. guachapele*, *T. rosea*, and *T. superba*) exposed to contrasting levels of cadmium (0, 6, and 12 ppm) for 90 days. Mean values ± standard error (*n* = 3). Different capital letters mean significant differences between plant species at the same contamination level, and different lowercase letters represent significant differences between cadmium treatments in the same plant species, according to Tukey’s test (*p* < 0.05).

To validate the effective phytoextraction capacity of the selected species, cadmium accumulation was evaluated in two ways: (1) as a function of its tissue content (mg kg^−1^ dry mass) and (2) as a function of accumulation over time and allocation to different parts of the plant (mg plant^−1^). In general, all forest species evaluated exhibited a high increase in cadmium content in leaves, stems, and roots as compared to the respective control treatments (Figure 3). In leaves, the species that most concentrated cadmium was *A. guachapele* (70.1 mg kg^−1^), followed by *T. rosea* (46.9 mg kg^−1^), *T. superba* (27.1 mg kg^−1^), and *C. pyriformis* (10.5 mg kg^−1^), under a 12 ppm level of contamination (Figure 3A). In the stem, *C. pyriformis* and *T. superba* were the species that presented higher cadmium concentrations (130.9 and 125.3 mg kg^−1^, respectively), followed by *T. rosea* (77.2 mg kg^−1^) and A. guachapele (49.4 mg kg^−1^) (Figure 3B). In the roots, in turn, *A. guachapele* stood out for its higher concentration of cadmium (817.2 mg kg^−1^) when exposed to 12 ppm of cadmium chloride, followed by *T. superba* (776.2 mg kg^−1^), *C. pyriformis* (363 mg kg^−1^), and *T. rosea* (281.2 mg kg^−1^) (Figure 3C).

Under 12 ppm Cd, all forest species studied here showed a tendency towards a higher concentration of Cd in leaves, except for *C. pyriformis*, which maintained this concentration stable at leaf level (Figure S4). Regarding the dynamics presented in the stem, additionally, *C. pyriformis* was the only species that showed a strong tendency to increase concentration over time, followed by *T. rosea*, which also showed a small increase in Cd concentration in the stem after 90 days of exposure. *A. guachapele* and *T. superba* under such conditions did not show cadmium concentration dynamics altered as a function of time, presenting a high concentration already at 30 days (Figure S5). Regarding cadmium dynamics in the roots, *C. pyriformis* and *A. guachapele* exhibited a concentration decrease after 60 days of exposure in both high cadmium conditions (Figure S6).

**Figure 4:**
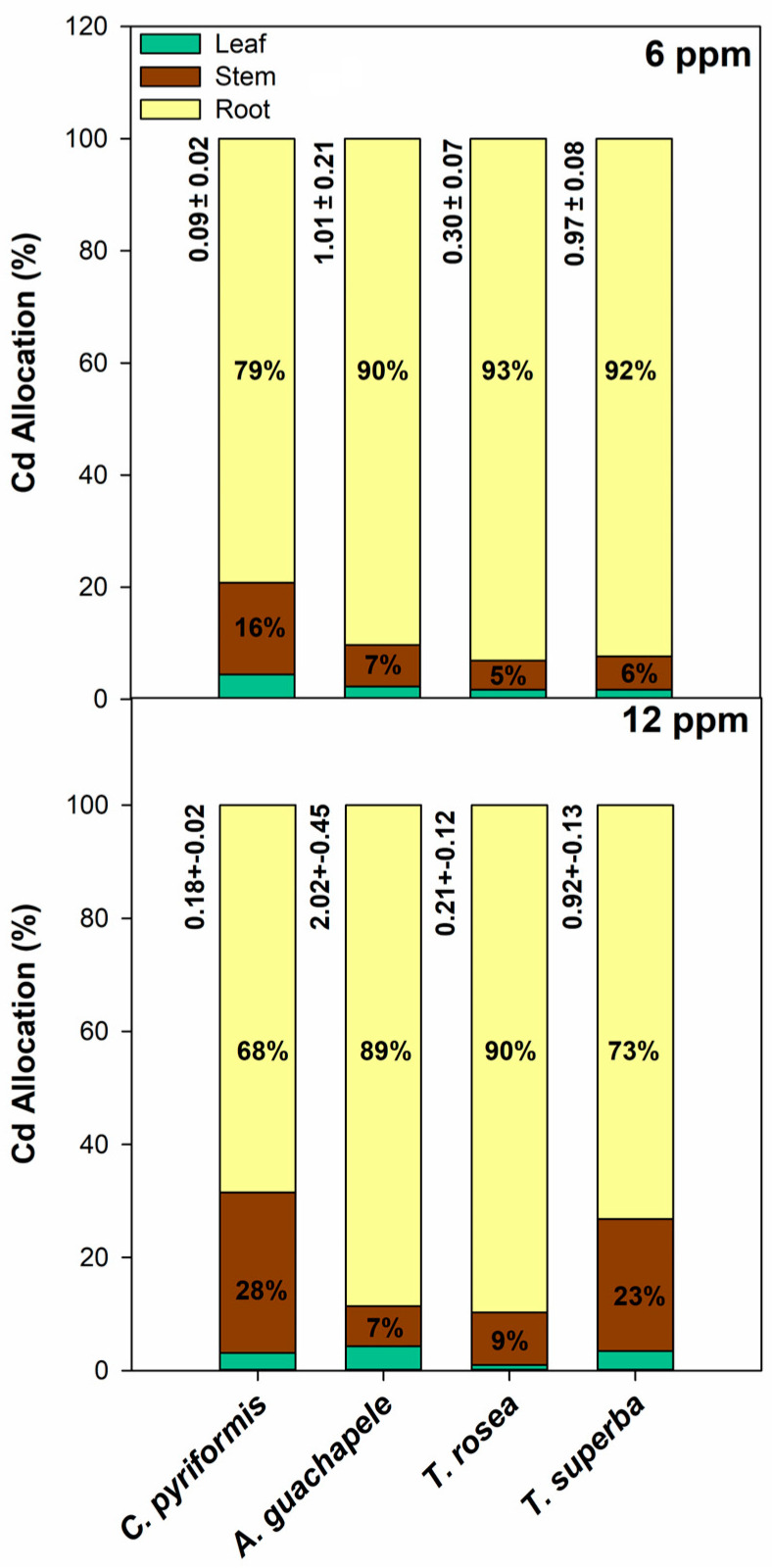
Cadmium allocation (%) in roots, stems, and leaves (mg cadmium plant^−1^) of plants of four forest species (*C. pyriformis*, *A. guachapele*, *T. rosea*, and *T. superba*) exposed to different levels of cadmium (6 and 12 ppm) for 90 days. Values on the left side of the bars indicate the total amount of cadmium uptake per plant unit (mg cadmium plant^−1^) after 90 days of exposure; mean values ± standard error (*n* = 3).

Considering that the cadmium treatment generates physiological alterations that may affect the growth and development of plants, the analysis based only on tissue concentration is not enough to generate a complete panorama that allows comparing the phytoextractor potential of each of the plant species since the base mass (DW) can vary strongly according to the effect of the Cd exposure itself (Figure 2). In this case, to better understand the processes of cadmium allocation between different tissues, the total amount of cadmium accumulated per plant was calculated as a percentage allocated to leaves, stems, and roots (Figure 4). For all the evaluated species, regardless of the contamination level (6 and 12 ppm) and exposure time (30, 60, and 90 days), cadmium allocation was always more related to roots than shoots (Figure 4 and Figure S7). In fact, the highest allocation of cadmium to shoot tissues was achieved by *T. rosea* at 60 days of exposure to 12 ppm Cd (49%, 25% in stems, and 24% in leaves). Regarding the features associated with phytoextraction, after 90 days of exposure to 12 ppm Cd, two species stood out for the greater allocation of this metal to the stem: *C. pyriformis* and *T. superba*, with 28% and 23% of the allocation, respectively (Figure 4). However, considering the very low total accumulation of cadmium in *C. pyriformis* (about 0.2 mg plant^−1^), *T. superba* stands out.

## 4. Discussion

In the present study, the phytoextraction capacity of four species of shade plants commonly used in cocoa agroforestry systems was evaluated. These plant species, in addition to providing natural shading for the cultivation of cacao, are appreciated for their potential wood production. Therefore, the stem is the main harvestable part of these trees, and the main objective evaluated in the present study was to define the potential of phytoextraction in the context of CAFS. Accordingly, *T. superba* pulls ahead because of its greater potential for cadmium allocation to the stem. Therefore, among the four evaluated species, *T. superba* has the best cadmium phytoextraction potential to be exploited in CAFS.

According to [11,21], metal hyperaccumulator plants are species that accumulate metals primarily in the shoots while maintaining low metal concentrations in the roots. The critical concentration expected for cadmium in the aerial part of a hyperaccumulator plant would be higher than 100 mg Cd kg^−1^ dry mass [11,21]. Considering the cadmium concentration threshold in shoots (>100 mg kg^−1^), *T. superba* clearly exceeds these values, indicating a possible phytoextraction potential for this species. Shoot Cd concentrations reached by *T. superba* in the present study are very similar to those exhibited by *Populus* spp. and *Salix* spp., two important phytoextractor species reported in the literature, under similar contamination conditions [24,27,32,33,34,47,48,49]. *Swietenia macrophylla* and *Morus alba* L. are also important phytoextractor tree species previously reported [50,51,52]. 

In the case of *Swietenia macrophylla*, 30 days of exposure to 15 mg Cd L^−1^ induced a concentration of 154 mg kg^−1^ in twigs [50], which was only slightly higher than the concentration shown here by *T. superba* in the stem (~130 mg kg^−1^). *Morus alba* L., a pioneering perennial woody plant that can grow in 145 ppm Cd-contaminated soils, extracts 1.92–7.89 g Cd every year per 1 ha [52]. In soils contaminated with up to 55 ppm Cd(NO_3_)2·4H_2_O, the maximum content of Cd in the stems of *M. alba* after 180 days of exposure did not exceed 30 mg kg^−1^ [51]. In parallel, different *Salix* spp. genotypes grown under flooded, 40 ppm CdCl_2_ contaminated soils, exhibited between 20 and 40 mg kg^−1^ Cd in the stems after 90 days of exposure [53]. These results indicate that *T. superba* exhibits cadmium hyperaccumulation capacity in stems similar to or much superior to that of most tree species traditionally considered good phytoextractors.

Phytoextraction’s desired characteristics in plants are high growth rate, high aboveground biomass, a deep and highly branched root system, and efficient uptake and translocation of heavy metals to the shoots [22,36,54,55]. Based on these indicators, the results obtained in the present study indicate that *C. pyriformis* possibly does not have the best characteristics to be considered a phytoextraction plant. In fact, although this species shows evidence for hyperaccumulation due to the high Cd concentration in shoots (especially stems), its growth rate is much lower than that shown by other species such as *T. superba* and *A. guachapele*. In fact, despite the strong decrease in growth shown by *A. guachapele* because of cadmium exposure, the total dry weight of this plant species was very high. This characteristic is intrinsic to *A. guachapele* ontogenesis and therefore should be considered a favorable factor in this species survival under adverse conditions such as high cadmium contamination. In line with this argument, *A. guachapele* was the species that most absorbed cadmium from the substrate, followed by *T. superba*.

The results involving the evaluation of the phytoextraction potential of *A. guachapele* and *C. pyriformis* allow us to make an important reflection about the best markers to select plants in CAFS and especially highlight the limitation of focusing only over Cd concentration on a dry mass basis, as usually performed in such studies. The mass of living tissues is fluctuating and responsive to the environment, varying because of the natural growing process but also being affected by the potential stress caused by heavy metal exposure [56]. Variations in cadmium concentration in plant tissues can be achieved both by the net accumulation of cadmium and by cadmium concentrations that also reflect a decrease in the plant’s dry mass. The net Cd accumulation is a consequence of the balance between Cd absorption, mainly from the soil [57], and the activation of exclusion mechanisms. On the other hand, a Cd content (mg kg^−1^) increase in plant tissues can be caused by a growth rate delay compared to optimal growth conditions or tissue abscission (mainly leaves), not necessarily involving a net accumulation of cadmium in the plant. 

In fact, when analyzing only the shoot Cd concentration data, *C. pyriformis* should stand out for the higher Cd concentration in the stem exhibited here. However, because *C. pyriformis* presents low growth rates, a very low Cd phytoextraction per plant is achieved by this species. The opposite is displayed by *A. guachapele*, which has the lowest concentrations of cadmium in the stem among the four species evaluated, but because it has higher growth over time, it shows a much higher amount of cadmium extracted per plant unit. In this context, Yin et al. [56] reported that *Salix integra* plants exposed to 25 ppm CdCl_2_ for up to 90 days exhibit approximately 55% of the total cadmium absorbed by plants allocated to aboveground tissues. Here, *T. superba* presented about 30% of the Cd allocated in stems and leaves, which also reinforces the capacity of this species to work as a good phytoextractor.

Leaf abscission is another important aspect to be considered here, as evidenced by the reduction of the total number of leaves after 90 days of cadmium exposure. This aspect was more marked in *A. Guachapele*, which was possibly associated with the high concentrations of leaf cadmium that took place in this species, indicating a possible toxicity symptom [58]. Leaf abscission can be interpreted as an episode of toxicity since it reduces the total light interception capacity and therefore compromises the plant’s photosynthetic potential. However, the reduction of the total leaf area also coincides with lower transpiration rates [59] and therefore lower passive transport of minerals (including cadmium) via xylem. In addition, the high accumulation of leaf toxic elements, especially in older and senescent leaves, may also represent an active cadmium exclusion mechanism, which would work in a similar way to those already reported for Na^+^ [60] and NH_4_^+^ [61] in other plant species.

Thus, despite the fact that *A. guachapele* could show a potential capacity for phytoextraction based on its high growth rates under cadmium contamination, this plant could negatively impact metal recycling due to its high rates of leaf senescence. In fact, *A. guachapele may* contribute 1780 tons ha^−1^ year^−1^ of litter to the soil [62]. Despite the great focus commonly given to the litter and fruit pods from cacao that are left on the farm after harvest, there have been no studies to date considering the effects on Cd deposition related to the senescent leaves from the CASF-associated trees [63,64,65]. Although these senescent leaves are of great importance for the cycling of carbon and mineral nutrients, the presumably increased Cd levels of some hyperaccumulating forest tree species may be an important issue. 

Controversial points such as the one related to metal cycling back to the soil through exacerbated leaf abscission in CAFS forest trees would justify recommending the use of herbaceous species for phytoextraction, neglecting the potential of forest trees in this role. Indeed, both herbaceous plants and trees have already been reported to have high potential for use in phytoextraction [66]. In their favor, herbaceous plants can achieve very high concentrations of cadmium in shoot tissues but produce very low total biomass, resulting in a low overall phytoextraction capacity. Hyperaccumulating trees, on the other hand, produce a very high mass, which can result in large amounts of cadmium being removed from the soil. However, low relative growth rates can result in very time-consuming phytoextraction processes, which implies a negative aspect [66]. 

The level of tolerance to the potential stress caused by exposure to the heavy metal is another important aspect to be considered here. According to Lux et al. [67], under 6 ppm Cd, *C. pyriformis*, *T. rosea*, and *T. superba* would be classified as highly tolerant (TI > 0.6), and *A. guachapele* would be among those with medium tolerance (TI > 0.35). Under 12 ppm, in turn, *C. pyriformis*, *T. superba*, and *A. guachapele* could be classified as having medium tolerance, and *T. rosea* would be the only species grouped as sensitive (TI < 0.35). On the one hand, high TI levels may reflect greater stability and capacity to maintain homeostasis [68], and plants can achieve this result through different physiological strategies.

*C. pyriformis* and *A. guachapele* present very close TI values under 12 ppm of Cd contamination (~0.5), but the total cadmium taken up by plant unit was almost 10 times higher in the latter one due to higher growth ratios. Tentatively, we could speculate here that *A. guachapele* presents a medium tolerance to cadmium through a possible hyperaccumulation (coping) mechanism, and *C. pyriformis* may present a similar medium tolerance to cadmium toxicity through a possible avoidance mechanism (low cadmium uptake). In fact, avoidance mechanisms can be triggered by many Cd-tolerant plants and may involve both the secretion of substances that favor the immobilization of the metal on the substrate [69] or down-regulation in the expression of metal transporters, such as NRAMP and the HMA family [70]. However, additional studies are still needed to understand the contrasting physio-genetic-molecular mechanisms in *C. pyriformis* under Cd exposure.

## 5. Conclusions

Taken together, the data obtained here allow us to conclude that *Terminalia superba* presents a more constant performance and is therefore identified with the greatest phytoextraction potential among the four forest species evaluated here. Indeed, considering all the favorable characteristics examined in context, Cd hyperaccumulation, high shoot Cd concentration, high tolerance index associated with high relative growth rates, low concentration of cadmium in leaves, and high allocation of cadmium to the stem (harvestable plant part as wood)—T. superba stands out as compared with the other CAFS species evaluated here or with the recognized phytoextractor tree species reported in the literature. *C. pyriformis* does not show solid evidence for a role in cadmium phytoextraction, and *T. rosea* shows strong evidence for being sensitive to this metal, especially at concentrations as high as 12 ppm. *A. guachapele*, in turn, presents high levels of Cd accumulation in plants over time but concentrates very high amounts of cadmium in the leaves. 

Nevertheless, additional studies at different stages of the plant development cycle under field conditions are still needed to determine the effective role of *T. superba* as phytoextractor plants in cacao agroforestry systems. Deepening this knowledge will be critical because it could help prevent food safety issues caused by excessive heavy metals (Cd) in cacao, a food or beverage ingredient highly consumed worldwide. 

## Figures and Tables

**Table 1:** Relative growth rate (RGR) of four forest species (*T. superba, A. guachapele, T. rosea, and C. pyriformis*) exposed to increasing levels of cadmium (6 and 12 ppm) for up to 90 days. The values are the mean ± SE (*n* = 3). Significant differences among treatments are represented by different letters. Duncan’s test, *p* < 0.05.

Treatment	Species	RGR (gg^−1^ day^−1^) (0–30 days)	RGR (gg^−1^ day^−1^) (30–60 days)	RGR (gg^−1^ day^−1^) (60–90 days)
0 ppm	*C. pyriformis*	0.006 ± 0.002 b	0.008 ± 0.001 b	0.012 ± 0.001 b
	*A. guachapele*	0.010 ± 0.002 ab	0.026 ± 0.009 a	0.030 ± 0.010 a
	*T. rosea*	0.012 ± 0.002 ab	0.013 ± 0.002 b	0.012 ± 0.005 b
	*T. superba*	0.018 ± 0.002 a	0.018 ± 0.002 a	0.025 ± 0.004 a
6 ppm	*C. pyriformis*	0.001 ± 0.002 b	0.001 ± 0.000 b	0.005 ± 0.001 b
	*A. guachapele*	0.012 ± 0.006 ab	0.011 ± 0.005 a	0.018 ± 0.006 a
	*T. rosea*	0.010 ± 0.002 ab	0.000 ± 0.000 b	0.010 ± 0.001 ab
	*T. superba*	0.020 ± 0.006 a	0.015 ± 0.004 a	0.017 ± 0.004 ab
12 ppm	*C. pyriformis*	0.000 ± 0.000 b	−0.001 ± 0.001 b	0.001 ± 0.002 b
	*A. guachapele*	0.012 ± 0.005 a	0.007 ± 0.004 a	0.022 ± 0.005 a
	*T. rosea*	−0.005 ± 0.002 b	−0.008 ± 0.002 b	−0.025 ± 0.015 b
	*T. superba*	0.017 ± 0.002 a	0.011 ± 0.002 a	−0.008 ± 0.003 b

## Data Availability

Data is unavailable due to privacy or ethical restrictions.

## Supplementary Materials

The following supporting information can be downloaded at: https://www.mdpi.com/article/10.3390/plants12162930/s1: Figure S1. Stem diameter in plants of four forest species (*C. pyriformis*, *A. guachapele*, *T. rosea*, and *T. superba*) exposed to contrasting levels of cadmium (6 and 12 ppm) for up to 90 days. Mean values ± standard error (*n* = 3). Asterisks represent significant differences between contrasting cadmium chloride treatments versus the control treatment within the same exposure time according to Tukey's test (*p* < 0.05). Figure S2. Plant height in four forest species (*C. pyriformis*, *A. guachapele*, *T. rosea*, and *T. superba*) exposed to contrasting levels of cadmium (6 and 12 ppm) for up to 90 days. Mean values ± standard error (*n* = 3). Asterisks represent significant differences between contrasting cadmium chloride treatments versus the control treatment within the same exposure time according to Tukey's test (*p* < 0.05). Figure S3. The number of leaves or leaflets in plants of four forest species (*C. pyriformis*, *A. guachapele*, *T. rosea*, and *T. superba*) exposed to contrasting levels of cadmium (6 and 12 ppm) for up to 90 days. Mean values ± standard error (*n* = 3). Asterisks represent significant differences between contrasting cadmium chloride treatments versus the control treatment within the same exposure time according to Tukey's test (*p* < 0.05). Figure S4. Cadmium content (mg cadmium ∙ kg^−1^ dry weight) in leaves of plants of four forest species (*C. pyriformis*, *A. guachapele*, *T. rosea*, and *T. superba*) exposed to contrasting levels of cadmium (6 and 12 ppm) for up to 90 days. Mean values ± standard error (*n* = 3). Asterisks represent significant differences between contrasting cadmium chloride treatments versus the control treatment within the same exposure time according to Tukey's test (*p* < 0.05). Figure S5. Cadmium content (mg cadmium ∙ kg^−1^ dry weight) in stem of plants of four forest species (*C. pyriformis*, *A. guachapele*, *T. rosea*, and *T. superba*) exposed to contrasting levels of cadmium (6 and 12 ppm) for up to 90 days. Mean values ± standard error (*n* = 3). Asterisks represent significant differences between contrasting cadmium chloride treatments versus the control treatment within the same exposure time according to Tukey's test (*p* < 0.05). Figure S6. Cadmium content (mg cadmium ∙ kg^−1^ dry weight) in roots of plants of four forest species (*C. pyriformis*, *A. guachapele*, *T. rosea*, and *T. superba*) exposed to contrasting levels of cadmium (6 and 12 ppm) for up to 90 days. Mean values ± standard error (*n* = 3). Asterisks represent significant differences between contrasting cadmium chloride treatments versus the control treatment within the same exposure time according to Tukey's test (*p* < 0.05). Figure S7. Cadmium allocation (%) in roots, stem, and leaves (mg cadmium ∙ plant^−1^) of plants of four forest species (*C. pyriformis*, *A. guachapele*, *T. rosea*, and *T. superba*) exposed to increased levels of cadmium (6 and 12 ppm) for 30 and 60 days. Values on the left side of the bars indicate the total amount of cadmium accumulated per plant (mean values ± standard error, *n* = 3).
