# Supplementary material for: Cadmium up Taking and Allocation in Wood Species Associated to Cacao Agroforestry Systems and Its Potential Role for Phytoextraction
Source: Plants (Basel). 2023 Aug 13;12(16):2930. doi: 10.3390/plants12162930 (PMC10459764; doi:10.3390/plants12162930)
Supplement: Supplementary file 1 [file plants-12-02930-s001.zip › plants-2509859-supplementary.pdf]

Supplementary material

Cadmium up taking and allocation in wood species associated to cacao agroforestry systems and its potential role for phytoextraction

Donald A. Galvis, Yeirme Y. Jaimes Suarez, Jairo Rojas Molina, Rosalba Ruiz, Fabricio E. L. Carvalho1*

2023

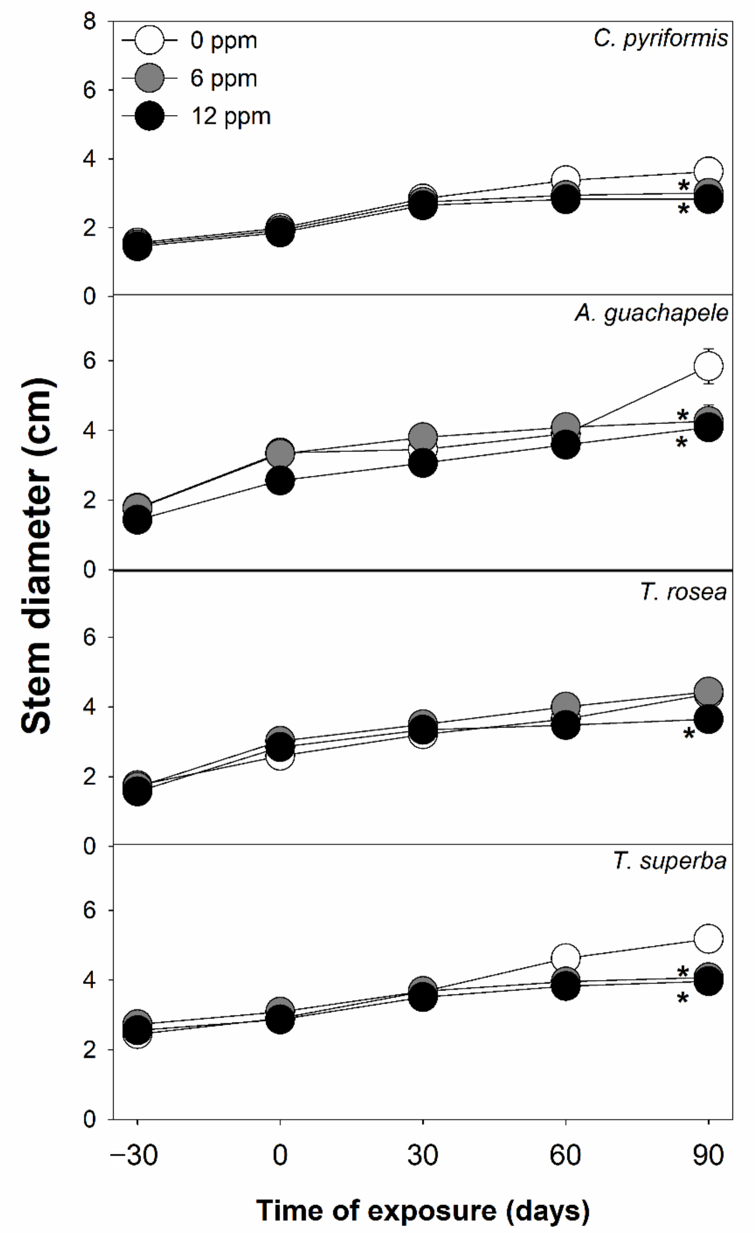

Figure S1. Stem diameter in plants of four forest species (*C. pyriformis*, *A. guachapele*, *T. rosea*, and *T. superba*) exposed to contrasting levels of cadmium (6 and 12 ppm) for up to 90 days. Mean values ± standard error (n=3). Asterisks represent significant differences between contrasting cadmium chloride treatments versus the control treatment within the same exposure time according to Tukey's test (p<0.05).

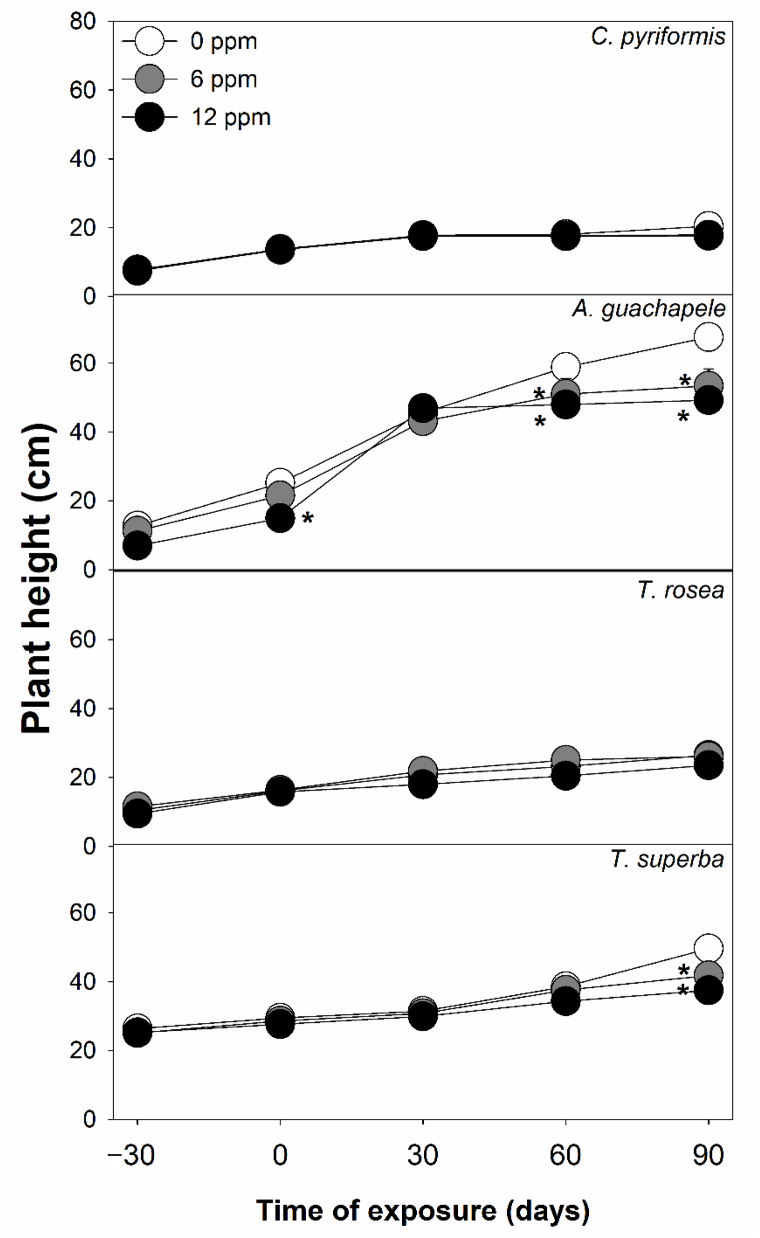

Figure S2. Plant height in four forest species (*C. pyriformis*, *A. guachapele*, *T. rosea*, and *T. superba*) exposed to contrasting levels of cadmium (6 and 12 ppm) for up to 90 days. Mean values ± standard error (n=3). Asterisks represent significant differences between contrasting cadmium chloride treatments versus the control treatment within the same exposure time according to Tukey's test (p<0.05).

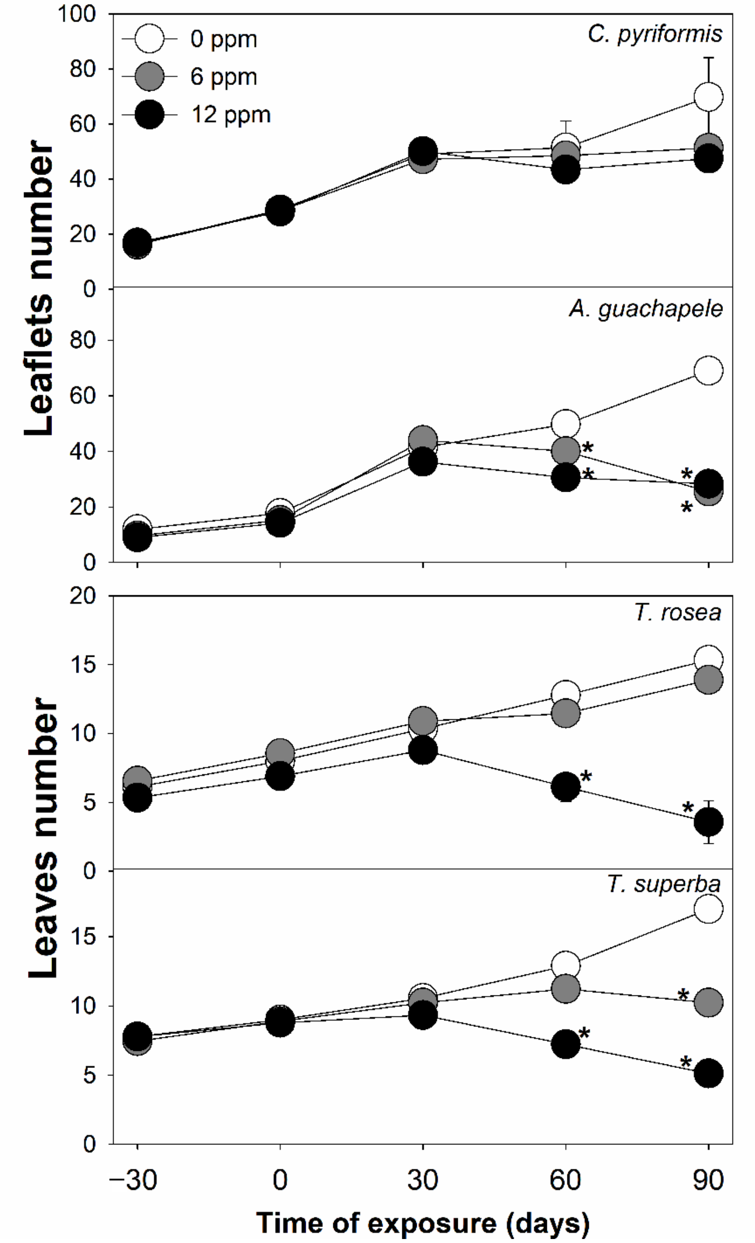

Figure S3. The number of leaves or leaflets in plants of four forest species (*C. pyriformis*, *A. guachapele*, *T. rosea*, and *T. superba*) exposed to contrasting levels of cadmium (6 and 12 ppm) for up to 90 days. Mean values ± standard error (n=3). Asterisks represent significant differences between contrasting cadmium chloride treatments versus the control treatment within the same exposure time according to Tukey's test (p<0.05).

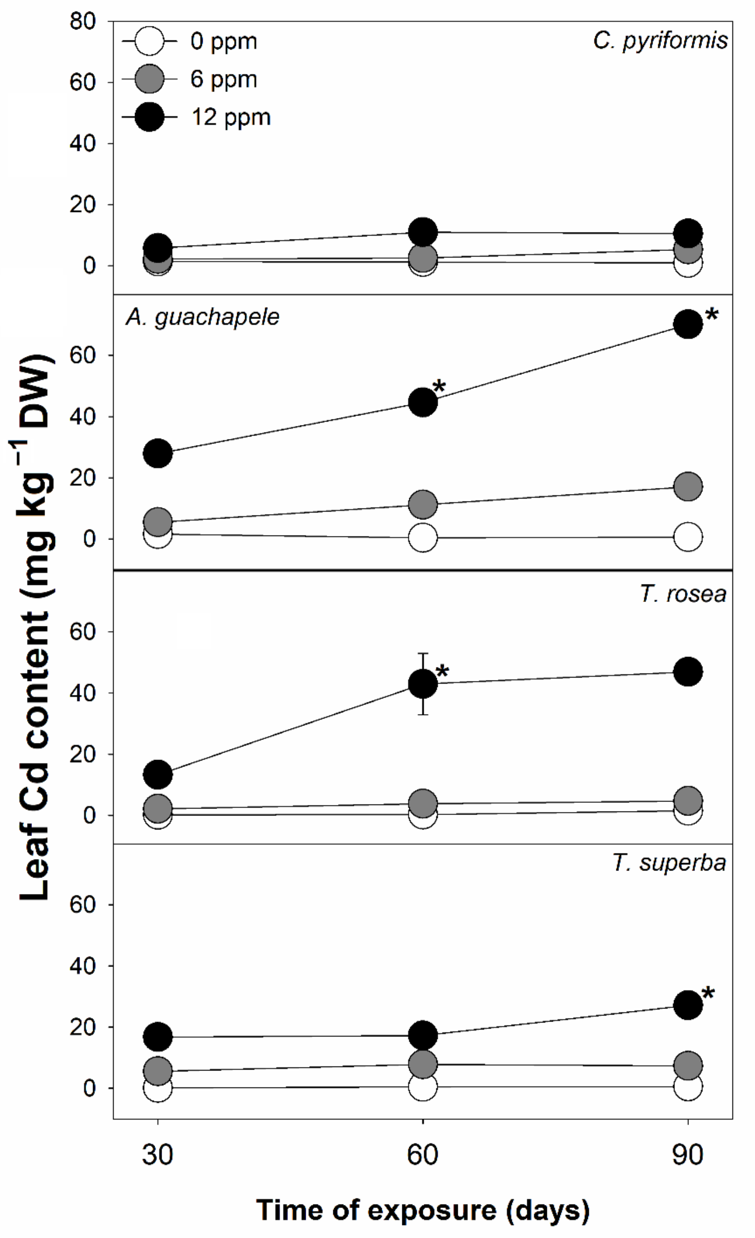

Figure S4. Cadmium content (mg cadmium · kg^{-1} dry weight) in leaves of plants of four forest species (*C. pyriformis*, *A. guachapele*, *T. rosea*, and *T. superba*) exposed to contrasting levels of cadmium (6 and 12 ppm) for up to 90 days. Mean values ± standard error (n=3). Asterisks represent significant differences between contrasting cadmium chloride treatments versus the control treatment within the same exposure time according to Tukey's test (p<0.05).

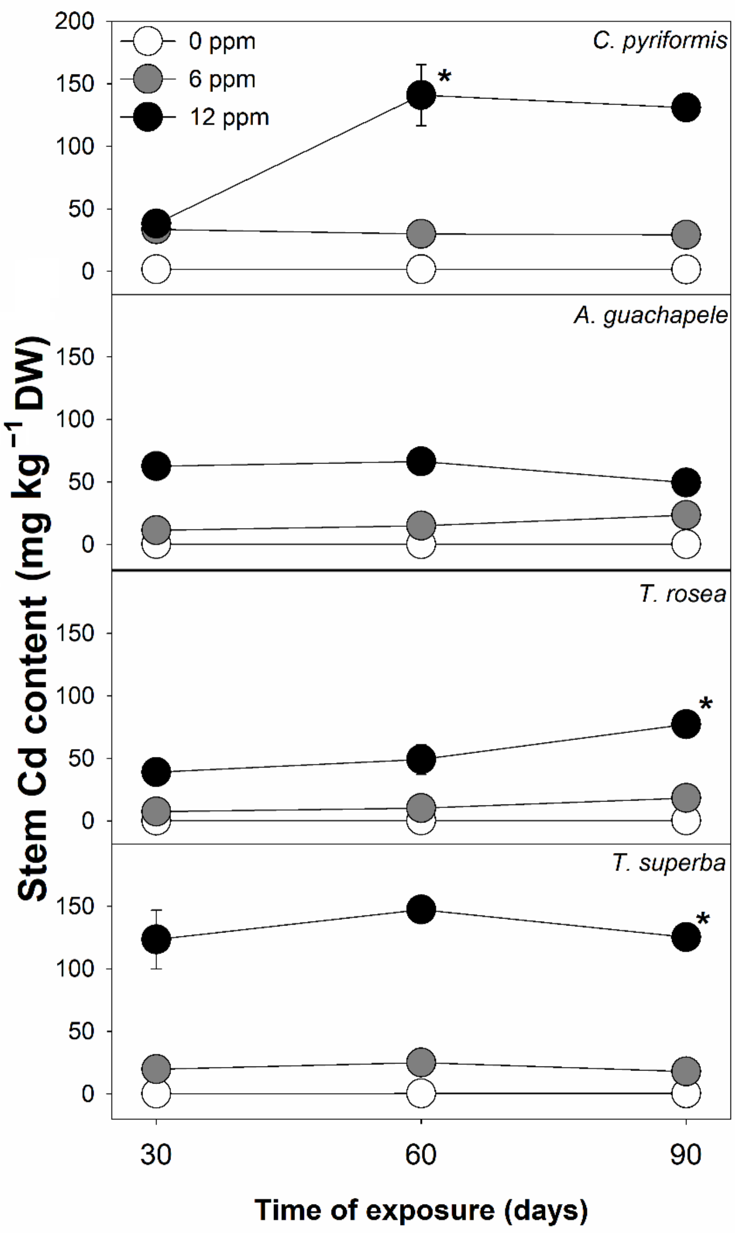

Figure S5. Cadmium content (mg cadmium · kg^{-1} dry weight) in stem of plants of four forest species (*C. pyriformis*, *A. guachapele*, *T. rosea*, and *T. superba*) exposed to contrasting levels of cadmium (6 and 12 ppm) for up to 90 days. Mean values ± standard error (n=3). Asterisks represent significant differences between contrasting cadmium chloride treatments versus the control treatment within the same exposure time according to Tukey's test (p<0.05).

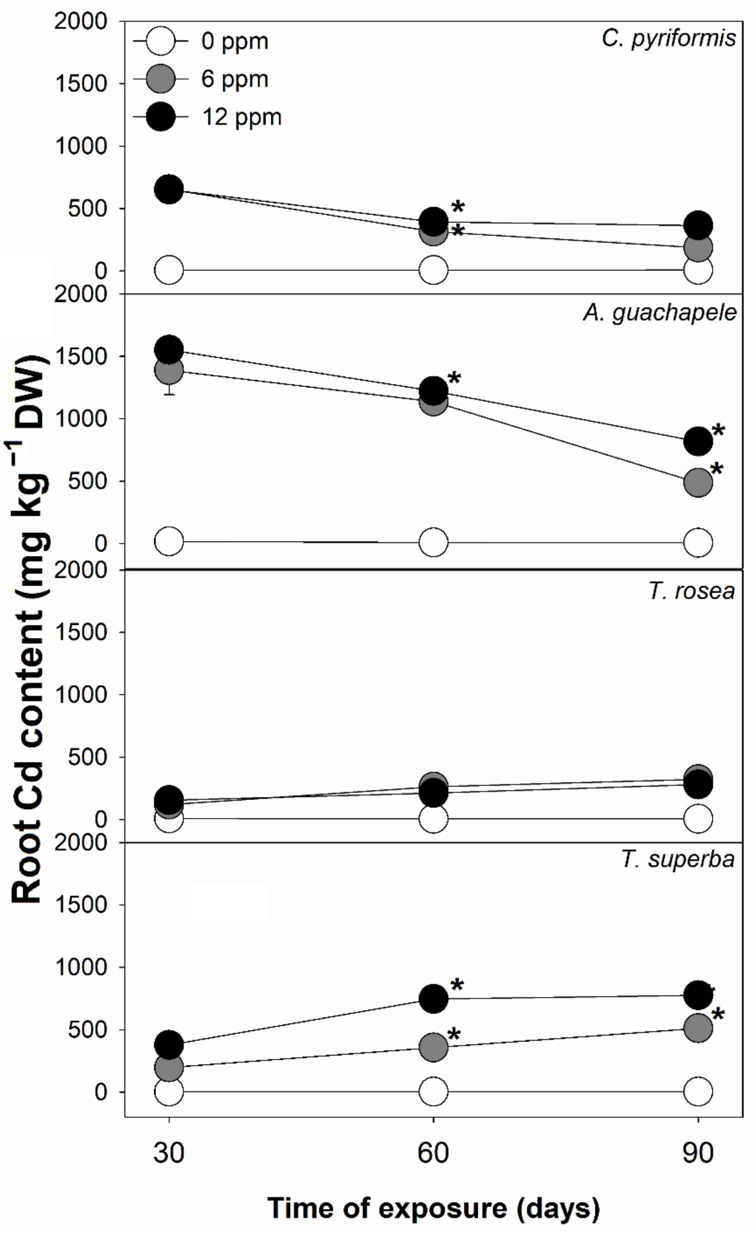

Figure S6. Cadmium content (mg cadmium · kg^{-1} dry weight) in roots of plants of four forest species (*C. pyriformis*, *A. guachapele*, *T. rosea*, and *T. superba*) exposed to contrasting levels of cadmium (6 and 12 ppm) for up to 90 days. Mean values ± standard error (n=3). Asterisks represent significant differences between contrasting cadmium chloride treatments versus the control treatment within the same exposure time according to Tukey's test (p<0.05).

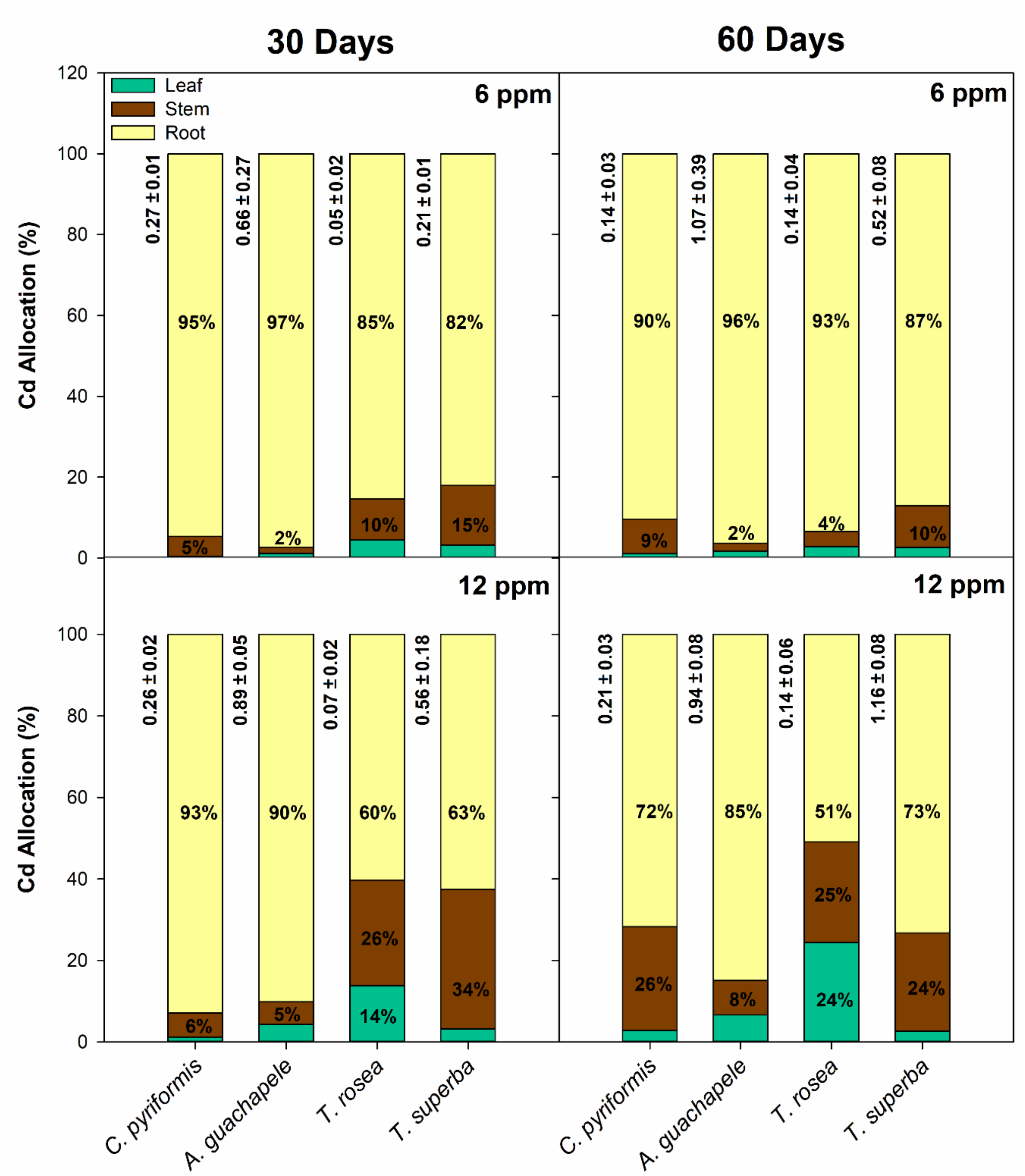

Figure S7. Cadmium allocation (%) in roots, stem, and leaves (mg cadmium · plant^{-1}) of plants of four forest species (*C. pyriformis*, *A. guachapele*, *T. rosea*, and *T. superba*) exposed to increased levels of cadmium (6 and 12 ppm) for 30 and 60 days. Values on the left side of the bars indicate the total amount of cadmium accumulated per plant (mean values ± standard error, *n*=3).
